# Caffeine Intake Mediates the Relationship Between Problematic Overstudying and Psychological Distress

**DOI:** 10.3390/nu17172845

**Published:** 2025-08-31

**Authors:** Oliwia Kosecka, Edyta Charzyńska, Stanisław K. Czerwiński, Agata Rudnik, Paweł A. Atroszko

**Affiliations:** 1 Institute of Psychology, University of Gdańsk, 80-309 Gdansk, Poland; o.kosecka.963@studms.ug.edu.pl (O.K.); stanislaw.czerwinski@ug.edu.pl (S.K.C.); agata.rudnik@ug.edu.pl (A.R.); 2 Institute of Psychology, Institute of Pedagogy, Faculty of Social Sciences, University of Silesia in Katowice, 40-126 Katowice, Poland

**Keywords:** study addiction, caffeine, stress, anxiety, depression

## Abstract

Background: Problematic overstudying has been conceptualized as an addictive behavior (study addiction) and an early form of work addiction. The majority of students showing compulsive studying behaviors experience chronic and high stress and symptoms of generalized anxiety disorder. Caffeine is a widely used stimulant that enhances alertness and cognitive performance, especially under fatigue. University students, particularly those exhibiting problematic overstudying, may consume more caffeine to improve academic performance. Previous research has shown that caffeine consumption is positively associated with perceived stress and anxiety. This study examined the mediating role of caffeine consumption in the relationship between problematic overstudying and psychological distress (perceived stress, anxiety, and depression) among university students. Methods: Sample 1 consisted of 436 university students, and Sample 2 included 3421 students. The Bergen Study Addiction Scale, Perceived Stress Scale-4, and a measure of average daily caffeine consumption were used. Results: Results showed that caffeine consumption partially mediated the relationship between problematic overstudying and perceived stress. Students who study compulsively tended to consume more caffeine, which was, in turn, associated with higher perceived stress. This finding was replicated across both samples, and in the second, larger sample, caffeine intake also mediated between problematic overstudying and anxiety and depression. Conclusions: Excessive caffeine use among students who manifest problematic overstudying may increase their risk of developing, or aggravate existing, symptoms of anxiety or mood disorders. Limiting caffeine intake and promoting healthy alternatives, such as rest and recovery, is recommended to support mental health in this population.

## 1. Introduction

Caffeine is the most widely used psychoactive stimulant in the world. Over 74% of adults in a representative sample of the U.S. reported drinking caffeinated beverages daily [1]. Caffeinated beverages are the most popular sources of caffeine intake [2]. While coffee is the primary source of daily caffeine intake for adults, it is also commonly consumed through soft drinks, tea, and energy drinks [3]. Caffeine enhances readiness and the ability to remain mentally alert after fatigue, as well as improves a wide array of basic cognitive functions, and enhances performance during fatiguing and cognitively demanding tasks [4,5,6]. As it improves attention, especially when alertness is low, it becomes popular among university students, who tend to consume energy drinks to boost their performance while studying [7,8]. A previous study showed that 78.5% of students consume caffeine to feel more awake and alert, and 30.8% do it specifically to improve concentration [2].

However, caffeine consumption acts directly on the physiological stress systems, specifically the Hypothalamic–Pituitary–Adrenal axis (HPA axis), increasing both adrenocorticotropin and cortisol secretion, which in turn potentiates both cardiovascular and neuroendocrine stress reactivity, thereby increasing overall stress [9,10]. Caffeine intake is also positively correlated with perceived stress levels and high dosages may trigger anxiety and panic attacks in vulnerable individuals [11,12,13]. Therefore, motivation to improve academic performance via sustained mental effort under high caffeine intake may lead to counterproductive results associated with high stress, including triggering or developing an anxiety disorder [14]. However, moderate doses of caffeine have positive effects on mood due to its energizing effect [4,5,15,16]. A positive mood also increases self-reported pain tolerance [17]. A previous study showed that although high scores of stress and anxiety were related to higher caffeine consumption, there was no such relation between caffeine consumption and depression [18]. In previous research, consumption of caffeine was even found to be associated with a reduced risk of depression [16].

The aim of this study was to investigate whether high caffeine consumption among students mediates between study addiction and stress, anxiety, and depression. Study addiction (or “problematic overstudying” or “compulsive studying behavior”) is characterized by excessive, compulsive studying, associated with impaired functioning in other life domains and various harms [19]. Addicted individuals tend to lose control over studying and be overly concerned with it [20]. Study addiction is associated with high absorption [21], characterized by a full focus on tasks and states of positive physiological and subjective arousal [22,23]. Because caffeine exhibits analogous effects, its consumption may act as an additional reinforcing factor within the ineffective stress-coping model of problematic overstudying [24,25]. From this perspective, caffeine consumption may act as a seemingly problem-oriented coping strategy to increase study effort and regulate mood by (i) intensifying the absorption, or (ii) helping to prolong it in the face of increasing fatigue, or (iii) enabling distraction from pain and/or increasing pain threshold. Below, a model explaining this mechanism is proposed, in which caffeine use during studying can be conceptualized as a form of functional “polydrug use”.

Theoretical and empirical evidence increasingly supports the conceptualization of problematic overstudying, so-called “study addiction”, as a behavioral addiction [24,25,26,27,28]. It has been identified as a potential early form of work addiction [29,30], a more established construct [17,26,31,32,33,34,35]. A recent cross-cultural longitudinal study provided evidence that study addiction and work addiction represent the same underlying addictive process with different manifestations at different stages of life and in different roles (student and working individual) [29,36].

Study addiction is associated with high and chronic stress which may lead to numerous well-documented health-related consequences [37]. Among them is educational burnout [38]. Moreover, study addiction is negatively associated with general quality of life, general health, sleep quality, and academic performance, as well as positively related to a host of psychopathological comorbidities, including anxiety and depression, social anxiety, eating disorders, and notably to obsessive compulsive personality disorder (OCPD) [19,24,25,26,27,28,39,40,41].

Cross-cultural research has consistently shown that the pleasure derived from learning is positively associated with study addiction [24,25]. In addition, negative affect and educational pressures serve as reinforcing factors [42]. It aligns with contemporary addiction theories that emphasize excessive goal-directed behavior (in the case of learning oriented toward reward-seeking outcomes such as academic achievement, social recognition, financial incentives, and the experience of competence) under conditions of negative emotional states as a central mechanism [43], and a significant role of environment [44].

Study addiction is associated with higher examination stress [45] and cardiovascular reactivity in situations related to being evaluated during studies [24]. Moreover, consumption of caffeinated beverages increases during academic stress [46]. Within the framework of the Transactional Theory of Stress and Coping [47], caffeine consumption and study addiction can be conceptualized as a coping response to perceived stressors. This assumption is consistent with previous findings that study addiction, like other addiction, is an ineffective coping mechanism and can be a way of managing negative emotions caused by examination stress through overstudying [24,48].

Associated with study addiction, absorption resembles the “high” state induced by substances such as stimulants [22,23]. Students become so deeply immersed in their studies that they lose awareness of their surroundings and struggle to disengage. This intense focus can become reinforcing, particularly for individuals facing personal or familial difficulties, as absorption allows them to avoid confronting distressing emotions, stress, or life challenges. Additionally, the physiological arousal resulting from intense study (associated with absorption) may obscure sensations such as fatigue or physical pain and discomfort, offering short-term relief [49]. However, it should be emphasized that the pain reduction effect may not be universal and constitute a specific risk factor for problematic overstudying among some individuals because stress-induced analgesia depends on age, gender, and prior experience with stressful, painful, or other environmental stimuli.

Caffeine also plays a role in modulating pain, and caffeine ingestion is associated with a higher pain threshold [50,51]. Randomized controlled trials [52] and a study on habitual caffeine intake substantiated caffeine’s analgesic effects [53]. In a study relevant to a typical studying context, participants who drank coffee before beginning a pain-inducing office task experienced less pain development than those who had not consumed coffee [54]. It is plausible that some of the mechanisms involved in caffeine’s analgesic synergy with nonsteroidal anti-inflammatory drugs (NSAIDs) [55], particularly adenosine receptor antagonism, may also contribute to the reduced pain experienced during mental or physical effort. However, this requires further research.

Caffeine use during studying can be conceptualized as a form of synergistic use or potentiation. This interaction between study behaviors and caffeine consumption may create a reinforcing loop, making the studying–caffeine dyad particularly effective in maintaining effort and escaping aversive internal states. In this sense, it may be considered a specific form of functional “polydrug” use, where a behavioral addiction (studying) is supported and sustained by a psychoactive substance (caffeine), contributing to the compulsive nature of the behavior and potentially increasing the risk of dependency-like patterns. Consequently, caffeine intake in study addiction may seem like a problem-focused coping strategy aimed to manage the stressor directly, or as an emotion-focused coping strategy to eliminate negative affect via cognitive distraction [56]. In fact, it is a maladaptive, addictive emotion regulation strategy [43].

Over time, this strategy of using absorption to regulate mood can lead to neural adaptations that cause discomfort in the absence of studying, manifesting in symptoms like restlessness or anxiety, typical of withdrawal. This sets up a self-perpetuating cycle. Excessive study leads to chronic stress and its consequences, including physical and mental health problems, which are then managed by further study. Paradoxically, similarly to study addiction, even though caffeine consumption can be a stress-coping mechanism, it may lead to higher stress and anxiety levels, sleep problems, and accumulating fatigue. When stress is combined with caffeine, it has an additive effect on stress hormones and blood pressure, meaning that caffeine intensifies the reaction to stressors [57].

High caffeine ingestion can lead to a condition known as “caffeinism,” characterized by symptoms such as restlessness, agitation, nervousness, irritability, anxiety, and heart palpitations [58,59]. The *Diagnostic and Statistical Manual of Mental Disorders* [60] and *International statistical classification of diseases and related health problems* [14] recognize a range of caffeine-related disorders, such as caffeine intoxication and caffeine withdrawal, that reflect the increasing clinical awareness of caffeine’s potential for misuse and its impact on health. Recognized by ICD-11, harmful caffeine use is a “pattern of continuous, recurrent, or sporadic use of caffeine that has caused clinically significant damage to a person’s physical health or mental health”.

Additionally, while some studies show potential negative health effects of caffeine consumption, it was previously argued that typically the role of high workload and compulsive work-related behaviors in extremely high caffeine consumption patterns is neglected [61]. High workload circumstances may serve as a confounding factor or rather a predisposing factor for high habitual caffeine intake. In this context, it is proposed that certain adverse effects linked to habitual high-dose caffeine consumption may be intricately connected to behaviors such as compulsive overstudying and overworking, with the underlying pathophysiological mechanisms primarily driven by workload-related stress (further increased and sustained by caffeine consumption) rather than caffeine per se [61].

**Figure 1:**
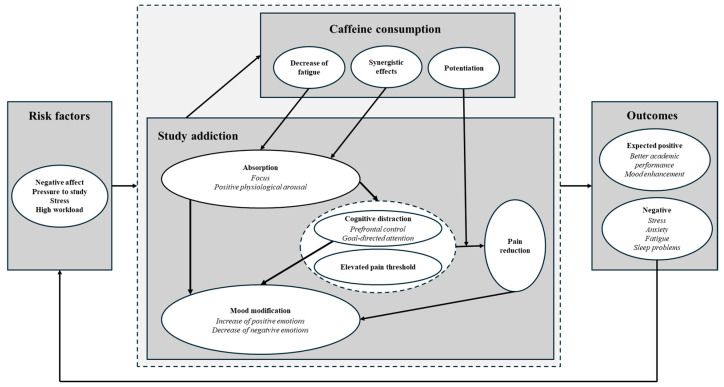
Model of study addiction and caffeine consumption as stress coping in response to negative affect, pressures to study, academic stress, and high workload. Study addiction is hypothesized to increase caffeine intake as an intentional, goal-oriented behavior to increase or prolong absorption (intense focus and positive physiological arousal), and in this way regulate mood. Specific intermediary mechanisms of cognitive distraction may serve this purpose by shifting attention from troubling thoughts and emotions, and together with an increased pain threshold associated with higher physiological arousal, it may (in some individuals) attenuate physical pain, which in turn may regulate mood. It is hypothesized that, paradoxically, this coping mechanism, instead of producing desirable results associated with increased productivity and enhanced mood, may lead to additional stress and anxiety, which in turn reinforce the loop and lead to increased study effort and caffeine consumption.

These mechanisms are illustrated in Figure 1, which presents a model explaining how study addiction and caffeine consumption may function as coping strategies. The model identifies relevant and specific risk factors for study addiction and caffeine intake, including negative affect [43], educational pressures [42], and high stress and workload [25,26].

While the model illustrates more detailed potential mechanisms of study addiction leading to high caffeine consumption and their interactions, as well as their determinants and consequences, the present study focuses on providing initial, robust evidence that this potential mechanism is feasible. If substantiated, further investigations into more detailed analyses of the model’s specific components (e.g., pain attenuation) should be conducted. Based on previous findings and theoretical frameworks, it is hypothesized that

**H1.**  *Study addiction is positively related to caffeine consumption.*

**H2.**  *Study addiction is positively related to perceived stress, anxiety, and depression.*

**H3.**  *Caffeine consumption is positively related to perceived stress and anxiety, but negatively to depression.*

**H4.**  *Caffeine consumption mediates the relationship between study addiction, perceived stress, anxiety and depression, and the indirect effect of study addiction on perceived stress and anxiety through caffeine consumption is positive, whereas the indirect effect on depression is negative.*

While within the presented model (Figure 1), feedback loops (with potential mediation effects) from stress to study addiction and caffeine consumption are plausible, the present study focuses on the initial mediation mechanism that seems to have the greatest practical relevance for two reasons. First, it suggests that anxiety and stress in addiction may be partly a result of caffeine consumption, making the potential reduction of its intake a feasible intervention. Second, it posits that study addiction leads to increased caffeine use, implying that preventing problematic overstudying and developing healthy study engagement instead could help minimize the harmful effects of caffeine use.

## 2. Materials and Methods

### 2.1. Participants

Sample 1 consisted of 436 university students: 340 females (80.8%), 81 males (18.6%), and 15 persons (3.4%) who chose the “other” option when responding to the question concerning gender, with the mean age of M = 21.98 years (SD = 2.98). Sample 2 consisted of 3421 university students: 2494 females (72.9%), 810 males (23.7%), and 117 persons (3.4%) who chose the “other” option, with the mean age of M = 21.75 years (SD = 3.07). In Sample 2, 3322 students were undergraduate or Master students, and 99 were Ph.D. students. The participants from both samples were students mainly from the University of Gdańsk, Poland. They participated in screening studies on the well-being of students conducted by a student research group in collaboration with the Academic Psychological Support Center at the University of Gdańsk.

### 2.2. Measures

Study addiction was assessed using the Bergen Study Addiction Scale (BStAS) [19], which includes seven items referring to experiences during the past 12 months (e.g., “How often during the last year have you studied in order to reduce feelings of guilt, anxiety, helplessness and depression?”). Each item is rated on a five-point Likert scale, ranging from never (1) to always (5). The Cronbach’s alpha reliability coefficient was 0.84 in Sample 1 and 0.82 in Sample 2. Study addiction symptoms were classified as present if the answer on the particular item measuring one of the common components of addiction from BStAS was 4 “often” or 5 “always” [28].

A single question regarding daily caffeine consumption was used: “How many cups of coffee and/or energy drinks do you consume per day (one cup is about 150 milliliters/one teaspoon of coffee/one can of energy drink is 250 milliliters)?” The participants were asked to answer with a single number, which was then treated as a number of “units” of caffeine. One teaspoon of coffee equals approximately 5 grams of ground or instant coffee. Self-report measures of caffeine intake demonstrated validity as a proxy for actual caffeine levels [62].

Stress was assessed using the Perceived Stress Scale (PSS-4) [63], which consists of four items (e.g., “In the last month, how often have you felt that you were unable to control the important things in your life?”). Each item is responded to on a five-point Likert scale, ranging from never (0) to very often (4). The Cronbach’s alpha reliability coefficient was 0.76 in Sample 1 and 0.78 in Sample 2.

Anxiety and depression were assessed by the Hospital Anxiety and Depression Scale (HADS) [64], consisting of 14 items: seven for anxiety (e.g., “I feel tense or ‘wound up’”), and seven for depression (e.g., “I feel as if I am slowed down”), each responded to on a four-point Likert response scale. The Cronbach’s alpha reliability coefficients were 0.77 for anxiety and 0.79 for depression in Sample 1, and 0.80 for anxiety and 0.76 for depression in Sample 2. In reviews of HADS performance, a cut-off score of ≥8 on either the HADS-A or HADS-D subscale is commonly used to identify possible cases of anxiety or depression, offering a favorable balance between sensitivity and specificity [65]. A higher threshold of ≥11 on HADS-A is often recommended to signal probable or definite anxiety, improving specificity though with reduced sensitivity—particularly useful in confirming diagnostic likelihood rather than broad screening [66]. In summary, a cut-off value of 8 or higher could be used to identify the risk of anxiety and depression, and a cut-off value of 11 or higher suggests clinically significant levels of anxiety and depression.

### 2.3. Procedure

The study was conducted in two stages. Sample 1 served as a pilot study, involving a smaller number of participants. It aimed to determine the feasibility of the basic hypothesized effects, particularly whether study addiction is associated with higher caffeine consumption, which is a basis for any further investigations. This was also important for estimating the initial effect sizes, particularly in the context of considerable measurement error in self-report dietary assessment data. Since the real effects of caffeine are likely to be underestimated, studies must comprise larger samples to obtain significant results. Based on this initial study, we determined whether it is feasible and meaningful to conduct further investigations, and we conducted a study in Sample 2, which was considerably larger, to provide greater statistical power to validate the patterns observed in the pilot.

Data were collected from April to June 2022 (Sample 1) and from March to July 2025 (Sample 2) using a convenience sampling method via an anonymous, voluntary online survey. Participants were provided with information about the study and its conditions, and their consent to participate was obtained. No monetary or other material rewards were offered.

### 2.4. Statistical Analyses

Descriptive statistics, means, standard deviations, percentages, and correlation coefficients were calculated. Estimates of prevalence were provided for anxiety and depression.

**Figure 2:**
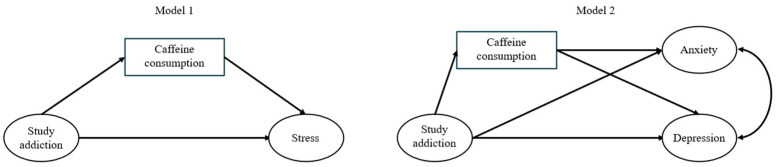
Path diagrams for the tested mediation models. Note: Control variables (gender and age), and measurement parts of the model for latent variables (study addiction, stress, anxiety, and depression) are omitted from the figure for clarity.

Missing data were handled using the full information maximum likelihood (FIML). Mediation models were tested using structural equation modeling (SEM) in MPlus 8.11 software [67]. In the first model, study addiction was the independent variable, caffeine consumption was the mediator, stress was the dependent variable, and the effects of gender and age were controlled for. In the second model, study addiction was the independent variable, caffeine consumption was the mediator, anxiety and depression were the dependent and correlated variables, and the effects of gender and age were controlled for. Study addiction, stress, anxiety, and depression were modeled as latent variables (consisting of all items on the scale used to measure the given variable), while caffeine consumption, gender, and age were treated as observed variables. Both models were tested in both samples and are presented in Figure 2.

For the model with stress as the dependent variable, the Robust Maximum Likelihood (MLR) estimator was used, while for the model with anxiety and depression as the dependent variables, the Weighted Least Squares Mean and Variance adjusted (WLSMV) estimator was used due to the HADS scale using a four-point response format [68]. The following measures were used to evaluate model fit: the Root Mean Squared Error of Approximation (RMSEA), the Comparative Fit Index (CFI), and the Standardized Root Mean Square Residual (SRMR). Cut-off scores for those indexes for an acceptable fit that were utilized were CFI ≥ 0.90, RMSEA ≤ 0.08, and SRMR ≤ 0.08 [69,70]. The 95% bias-corrected confidence intervals were calculated from 5000 bootstrap replicates.

## 3. Results

The overall percentage of missing values was 1.96% in Sample 1 and 0.61% in Sample 2. Table 1 presents mean scores, standard deviations, percentages, and correlation coefficients of the study variables. In both samples, study addiction correlated positively with caffeine consumption, perceived stress, anxiety, and depression.

Table 2 presents caffeine intake, stress levels, and the prevalence of anxiety and depression by number of study addiction symptoms. The presence of study addiction symptoms was associated with fewer students who do not consume caffeine at all and a higher percentage of participants who consume more than six units of caffeine a day in comparison to students showing no study addiction symptoms. Students who showed all seven study addiction symptoms in comparison to students showing no study addiction symptoms had from over two to over three times higher rates of clinical depression and anxiety, depending on the sample.

Table 3 presents model fit indices for both mediation models. All tested models fit the data well. Caffeine consumption partially mediated the relationship between study addiction and stress in both samples (see Table 4). The relationship between study addiction and anxiety, as well as the relationship between study addiction and depression, was partially mediated by caffeine consumption in Sample 2, but not in Sample 1 (see Table 5).

## 4. Discussion

Study addiction was positively related to caffeine consumption (H1 substantiated), meaning that individuals addicted to studying tend to drink more coffee and/or energy drinks. Higher caffeine intake appears to be a goal-oriented behavior and coping strategy [47], as it enables students to study more by improving attention, reducing fatigue, and pain [8,54], as well as distracting from problems and enhancing mood. However, in the context of problematic overstudying, it constitutes an ineffective and addictive pattern of mood regulation [24,25].

The results supported previous findings that study addiction is positively related to perceived stress, anxiety, and depression (H2 substantiated) [41]. Caffeine consumption was positively related to perceived stress in both samples, but only in Sample 2 to anxiety and depression (H3 partially substantiated). Importantly, the association between caffeine consumption and depression was positive, which was not expected. Consequently, the indirect effect was in the same direction as in the case of stress and anxiety. The relationship between caffeine consumption and anxiety, and between caffeine consumption and depression in Sample 1, may not have been significant due to the smaller size of this sample and, therefore, insufficient statistical power. Dietary measurement error usually causes associations to be underestimated, so studies must comprise larger samples to compensate for measurement imprecision [71]. However, those results should still be interpreted carefully, and more studies regarding this topic are needed.

The results from Sample 2, indicating that caffeine intake is positively related to anxiety, are consistent with previous findings [72]. However, meta-analyses show a negative association between caffeine consumption and depression risk [16,73,74]. Yet, a recent study showed that higher caffeine intake was associated with an increased risk of suicidal ideation among shift workers [75]. It was suggested that it is due to long working hours and sleep deprivation, which is associated with high caffeine consumption [76]. Decreased sleep increases the risk of depression [77]. Therefore, accordingly to what was previously argued, very high caffeine consumption may be a confounding or mediating factor in the association of high workload, long working hours, and job stress with health outcomes [61]. In the present study, the positive association between caffeine consumption and depression may be partially explained by similar mechanisms associated with high study workload, long studying hours, and high academic stress related to study addiction [25].

Caffeine consumption partially mediated the relationship between study addiction and perceived stress in both samples. However, it was found to be the partial mediator of the relationship between study addiction and anxiety, and between study addiction and depression, only in Sample 2 (H4 partially substantiated). Those mediations could be insignificant in Sample 1 due to insufficient statistical power resulting from the small sample size, as the effect sizes were similar in both samples. Therefore, the findings from Sample 1 should be interpreted as preliminary and limited by sample size. Caffeine consumption mediates these relationships only partially and the effects are small, as several other mechanisms (e.g., excessive efforts, perfectionism, social isolation) may also contribute to higher levels of perceived stress, anxiety, and depression in addicted individuals [41]. Moreover, the association of these outcome variables with caffeine consumption is likely underestimated due to considerable measurement error. As caffeine is used to cope with high workload in students, its reinforcing effect on study addiction may outweigh its mood enhancement properties, leading to an increased risk of depression, as well as generalized anxiety disorder or likely panic disorder.

### Strengths, Limitations, and Future Directions

To the authors’ knowledge, this is the first study to investigate the relationship between study addiction and caffeine consumption, and their associations with stress, anxiety, and depression. It was based on two relatively large sample sizes, especially Sample 2, allowing for meaningful analyses with proper statistical power, especially in the context of nutritional epidemiology, which typically requires very large sample sizes to compensate for measurement imprecision and numerous confounding factors. Moreover, the results were mostly replicated, providing more robust support for the feasibility of the proposed theoretical model. Valid and reliable measures of variables in this study were used. Consequently, the study significantly adds to the existing literature on both study addiction and caffeine consumption, as well as behavioral addictions and substance use generally.

In terms of limitations, the results of this study cannot be generalized to other populations without restrictions due to the non-representative sample. Moreover, all data were self-reported. Reliable psychometric measures were used to assess anxiety and depression, but they cannot substitute for a clinical diagnosis. Self-report dietary assessment data have important limitations in interpreting results due to measurement error, especially recall bias and underestimation of negative substance intake [71]. Moreover, this study did not include sources of caffeine other than coffee and energy drinks. Additionally, the content of caffeine in different brands of coffee and energy drinks varies greatly. Although caffeine is ingested mainly via drinking coffee, excluding its other sources (e.g., tea) or the brewing method may obscure potential associations in studies examining coffee as a risk factor for disease occurrence [78].

Furthermore, although mediation analyses were conducted, the cross-sectional study design cannot be treated as compelling evidence of causal relationships between variables. It is likely that alternative models, particularly representing potential feedback loops, may also fit the data. Nevertheless, the mediation model in the present study is strongly substantiated by the feasible causal mechanisms based on theoretical assumptions, particularly addiction theory and stress coping, and previous studies. It also addresses the most practically significant mechanism from the point of view of harm reduction and prevention solutions.

In this context, the current study paves the way for systematic investigations of mechanisms associated with caffeine intake and its health effects in addictive disorders associated with overworking and overstudying, as well as generally in association with high workload and occupational and academic stress. The suggested model (Figure 1) can be investigated in more detail with longitudinal studies, particularly intensive longitudinal designs such as ecological momentary assessment (EMA), experience sampling method (ESM), daily diary studies, ambulatory physiological monitoring, or even with experimental designs. These would provide more robust evidence regarding the directionality of the relationships among the variables. Moreover, such designs would allow for investigation of nuanced postulated (in the model) mechanisms associated with synergistic effects or potentiation associated with study addiction and caffeine intake interactions. These effects require more precise control over caffeine intake and dosing, and direct measurement of its impact on affective states, cognitive functioning, and performance, as well as potential health consequences, especially acute ones such as sleep disturbances. In future studies, genetic differences should also be taken into consideration, as they change individuals’ reactions to caffeine. Specifically, CYP1A2 encodes the main liver enzyme metabolizing caffeine, and its variants determine whether someone is a fast or slow caffeine metabolizer [79].

## 5. Conclusions

In this article, a theoretical model of study addiction and caffeine intake as a stress-coping mechanism is proposed. It suggests a specific form of “polydrug use” in which behavioral addiction is reinforced by substance use. The present study provides initial support for the plausibility of this model. Caffeine consumption mediated the relationship between study addiction and perceived stress, anxiety, and depression. Problematic overstudying is associated with higher caffeine consumption, as caffeine becomes a tool to study longer and be more productive. The findings suggest that, as it reinforces the pathological mechanism of study addiction and increases physiological arousal, it increases the mental and physical health risks already associated with study addiction, and mediated by stress. These include anxiety and depressive disorders. Vulnerable groups, including children, adolescents, pregnant individuals, and those with caffeine sensitivity, should be advised to limit their consumption to minimize potential health risks. This is particularly important, as study addiction is relatively prevalent among high school students [80], and energy drinks, despite being illegal for minors to consume in many countries, tend to be relatively commonly consumed by adolescents [81]. Since caffeine appears to reinforce problematic overstudying, limiting its intake may help reduce some of the harmful consequences of study addiction and somewhat alleviate some of its symptoms, though it is unlikely to resolve study addiction itself.

Prevention and harm reduction solutions should encourage healthy alternatives, such as rest and recovery, and effective emotion regulation strategies. Effective strategies could involve the cultivation of social support networks, maintaining a balanced lifestyle with proper sleep and nutrition, and practicing mindfulness or other stress-management techniques. Given the associations between problematic overstudying, caffeine use, and mental health symptoms, universities and public health institutions could consider targeted interventions. These may include implementing psychoeducational programs about healthy study habits and caffeine risks, as well as integrating these topics into student well-being curricula. The findings should also be interpreted in light of the broader sociocultural context that values productivity, academic excellence, and ongoing self-improvement. This performance-driven culture may normalize or even reward maladaptive coping mechanisms, including study addiction and high caffeine use. Interventions should thus consider not only individual behavior change but also systemic academic reforms aimed at reducing performance pressure. The potential mechanisms underlying study addiction leading to high caffeine consumption, including absorption, cognitive distraction, mood modification, and pain reduction, should be investigated in future studies, particularly in intensive longitudinal and experimental designs.

## Figures and Tables

**Table 1:** Mean scores, standard deviations (SD), percentages, and correlation coefficients of the study variables.

	Mean (SD)								
Variable	Sample 1	Sample 2	1.	2.	3.	4.	5.	6.	7.
1. Study addiction	20.26 (6.08)	18.66 (5.79)	-	0.11 **	0.30 **	0.44 **	0.28 **	−0.21 **	−0.02
2. Caffeine consumption	1.62 (1.73)	1.56 (1.56)	0.20 **	-	0.08 **	0.15 **	0.14 **	0.02	0.12 **
3. Stress	12.73 (3.11)	12.19 (3.13)	0.28 **	0.17 **	-	0.62 **	0.60 **	−0.13 **	−0.02
4. Anxiety	12.05 (4.20)	11.00 (4.39)	0.44 **	0.16 **	0.62 **	-	0.54 **	−0.20 **	0.00
5. Depression	7.70 (3.84)	6.81 (3.87)	0.21 **	0.09	0.58 **	0.52 **	-	−0.01	0.03 *
6. Gender	80.8% female	72.9% female	−0.17 **	−0.08	−0.17 **	−0.25 **	−0.13 **	-	0.04 *
7. Age	21.98 (2.98)	21.75 (3.07)	−0.03	0.10 *	−0.07	−0.07	−0.01	0.07	-

* *p* < 0.05. ** *p* < 0.01. Gender was dummy-coded (0 = female, 1 = male). Due to the low number of participants who chose the option ‘Other,’ they were not included in the analyses. Note: Below the diagonal are correlations for Sample 1, and above the diagonal are correlations for Sample 2.

**Table 2:** Caffeine intake, stress levels, and the prevalence of anxiety and depression by number of study addiction symptoms.

Number of Study Addiction Symptoms	n (%) of Individuals	Caffeine Units Consumed per Day M (SD)	n (%) of Individuals Who Do Not Consume Caffeine	n (%) of Individuals Consuming ≥ 6 Units of Caffeine per Day	Stress M (SD)	Mild Anxiety n (%)	Clinical Anxiety n (%)	Mild Depression n (%)	Clinical Depression n (%)
Sample 1 (N = 436)									
0	79 (18.1%)	1.25 (1.33)	33 (41.8%)	0 (0%)	11.55 (3.32)	58 (73.4%)	29 (36.7%)	25 (31.6%)	12 (15.2%)
1	80 (18.3%)	1.45 (1.34)	23 (28.7%)	0 (0%)	11.95 (3.06)	55 (68.8%)	36 (45.0%)	31 (38.8%)	11 (13.8%)
2	69 (15.8%)	1.42 (1.38)	22 (31.9%)	1 (1.4%)	12.63 (3.12)	61 (88.4%)	42 (60.9%)	37 (53.6%)	20 (29.0%)
3	63 (14.5%)	1.52 (1.39)	18 (28.6%)	0 (0%)	13.13 (2.72)	60 (95.2%)	51 (81.0%)	38 (60.3%)	22 (34.9%)
4	56 (12.9%)	2.02 (1.80)	12 (21.4%)	2 (3.6%)	13.67 (2.97)	55 (98.2%)	46 (82.1%)	29 (51.8%)	15 (26.8%)
5	42 (9.6%)	1.67 (1.39)	13 (31.0%)	0 (0%)	12.90 (2.82)	39 (92.9%)	30 (71.4%)	22 (52.4%)	11 (26.2%)
6	29 (6.7%)	2.48 (3.68)	7 (24.1%)	1 (3.4%)	13.36 (2.91)	28 (96.6%)	23 (79.3%)	18 (62.1%)	10 (34.5%)
7	18 (4.1%)	2.44 (2.06)	3 (16.7%)	2 (11.1%)	15.47 (2.50)	18 (100.0%)	17 (94.4%)	13 (72.2%)	7 (38.9%)
Total sample	436 (100%)	1.62 (1.73)	131 (30.0%)	6 (1.4%)	12.73 (3.11)	374 (85.8%)	274 (62.8%)	213 (48.9%)	108 (24.8%)
Sample 2 (N = 3421)									
0	904 (26.4%)	1.31 (1.47)	328 (36.3%)	14 (1.5%)	10.94 (3.15)	499 (55.2%)	260 (28.8%)	236 (26.1%)	99 (11.0%)
1	693 (20.2%)	1.46 (1.52)	226 (32.6%)	7 (1.0%)	11.85 (2.99)	488 (70.4%)	312 (45.0%)	234 (33.8%)	114 (16.5%)
2	615 (18.0%)	1.59 (1.56)	173 (28.1%)	15 (2.4%)	12.45 (3.00)	500 (81.3%)	362 (58.9%)	238 (38.7%)	111 (18.0%)
3	443 (12.9%)	1.79 (1.61)	113 (25.5%)	12 (2.7%)	12.58 (2.94)	384 (86.7%)	283 (63.9%)	199 (44.9%)	85 (19.2%)
4	307 (9.0%)	1.78 (1.66)	82 (26.7%)	11 (3.6%)	13.10 (2.80)	282 (91.9%)	225 (73.3%)	166 (54.1%)	73 (23.8%)
5	218 (6.4%)	1.79 (1.52)	48 (22.0%)	5 (2.3%)	13.72 (2.76)	213 (97.7%)	177 (81.2%)	141 (64.7%)	64 (29.4%)
6	164 (4.8%)	1.80 (1.61)	37 (22.6%)	5 (3.0%)	13.97 (2.79)	158 (96.3%)	144 (87.8%)	98 (59.8%)	60 (36.6%)
7	77 (2.3%)	1.81 (1.65)	21 (27.3%)	2 (2.6%)	13.71 (2.73)	75 (97.4%)	69 (89.6%)	54 (70.1%)	28 (36.4%)
Total sample	3421 (100%)	1.56 (1.56)	1028 (30.0%)	71 (2.1%)	12.19 (3.13)	2599 (76.0%)	1832 (53.6%)	1366 (39.9%)	634 (18.5%)

Note: M = mean, SD = standard deviation.

**Table 3:** Model fit indices for the mediation models.

Dependent Variables	Sample	χ^2^	*df*	CFI	RMSEA	90% CI	SRMR
Stress	Sample 1	193.07	69	0.921	0.064	[0.054–0.075]	0.053
	Sample 2	730.46	69	0.944	0.053	[0.050–0.056]	0.036
Anxiety and depression	Sample 1	578.75	240	0.913	0.054	[0.048–0.060]	0.057
	Sample 2	2438.29	240	0.935	0.052	[0.050–0.054]	0.042

Note. CFI = Comparative Fit Index; RMSEA = Root Mean Square Error of Approximation; SRMR = Standardized Root Mean Squared Residual.

**Table 4:** Path analysis for the mediation models with stress as the dependent variable with 95% confidence intervals and standardized coefficients.

	Sample 1		Sample 2	
	β	95% CI	β	95% CI
*Direct effects*				
Study addiction → caffeine consumption	0.209 **	[0.103; 0.297]	0.135 **	[0.096; 0.172]
Caffeine consumption → stress	0.184 **	[0.072; 0.285]	0.059 **	[0.019; 0.100]
Study addiction → stress	0.319 **	[0.187; 0.443]	0.355 **	[0.313; 0.396]
*Indirect effect*				
Study addiction → caffeine consumption → stress	0.038 **	[0.014; 0.070]	0.008 **	[0.003; 0.014]
*Total effect*				
Study addiction → stress	0.357 **	[0.228; 0.478]	0.363 **	[0.322; 0.403]
*Effects of control variables*				
Gender → caffeine consumption	−0.047	[−0.133; 0.043]	0.044 *	[0.006; 0.083]
Gender → stress	−0.075	[−0.195; 0.044]	−0.087 **	[−0.125; −0.047]
Age → caffeine consumption	0.107 *	[0.022; 0.200]	0.120 **	[0.088; 0.155]
Age → stress	−0.067	[−0.186; 0.058]	−0.019	[−0.059; 0.019]

* *p* < 0.05. ** *p* < 0.01.

**Table 5:** Path analysis for the mediation models with anxiety and depression as the dependent variables with 95% confidence intervals.

	Sample 1		Sample 2	
	β	95% CI	β	95% CI
*Direct effects*				
Study addiction → caffeine consumption	0.241 **	[0.129; 0.324]	0.138 **	[0.098; 0.175]
Caffeine consumption → anxiety	0.034	[−0.068; 0.135]	0.090 **	[0.054; 0.127]
Caffeine consumption → depression	0.038	[−0.077; 0.142]	0.097 **	[0.058; 0.135]
Study addiction → anxiety	0.496 **	[0.389; 0.597]	0.499 **	[0.464; 0.532]
Study addiction → depression	0.273 **	[0.132; 0.382]	0.370 **	[0.328; 0.411]
*Indirect effects*				
Study addiction → caffeine consumption → anxiety	0.008	[−0.017; 0.036]	0.012 **	[0.007; 0.019]
Study addiction → caffeine consumption → depression	0.009	[−0.019; 0.037]	0.013 **	[0.008; 0.020]
*Total effects*				
Study addiction → anxiety	0.504 **	[0.401; 0.579]	0.511 **	[0.477; 0.544]
Study addiction → depression	0.283 **	[0.150; 0.391]	0.383 **	[0.342; 0.424]
*Effects of control variables*				
Gender → caffeine consumption	−0.037	[−0.124; 0.053]	0.047 *	[0.009; 0.085]
Gender → anxiety	−0.194 **	[−0.291; −0.099]	−0.120 **	[−0.154; −0.085]
Gender → depression	−0.120 *	[−0.225; −0.009]	0.056 **	[0.017; 0.094]
Age → caffeine consumption	0.110 *	[0.023; 0.202]	0.122 **	[0.089; 0.156]
Age → anxiety	−0.034	[−0.142; 0.074]	0.006	[−0.029; 0.041]
Age → depression	−0.003	[−0.114; 0.109]	0.041 *	[0.005; 0.077]

* *p* < 0.05. ** *p* < 0.01.

## Data Availability

The data supporting the results of this study will be available upon request from interested researchers. Further information and materials necessary for the reproduction of the experiment can be obtained by contacting the authors.

## Abbreviations

The following abbreviations are used in this manuscript:

HPA	Hypothalamic–Pituitary–Adrenal
OCPD	Obsessive Compulsive Personality Disorder
NSAIDs	Nonsteroidal anti-inflammatory drugs
cGMP	Cyclic guanosine monophosphate
BStAS	Bergen Study Addiction Scale
PSS-4	Perceived Stress Scale
HADS	Hospital Anxiety and Depression Scale
SEM	Structural equation modeling
MLR	Robust Maximum Likelihood
WLSMV	Weighted Least Square Mean and Variance adjusted
RMSEA	Root Mean Squared Error of Approximation
CFI	Comparative Fit Index
SRMR	Standardized Root Mean Square Residual
EMA	Ecological Momentary Assessment
ESM	Experience Sampling Method
